# Feasibility Trial Exploring Immune-Related Biomarkers Pertaining to Rapid Immune Surveillance and Cytokine Changes after Consuming a Nutraceutical Supplement Containing Colostrum- and Egg-Based Low-Molecular-Weight Peptides

**DOI:** 10.3390/cimb46070400

**Published:** 2024-06-30

**Authors:** Liu Yu, Ifeanyi Iloba, Dina Cruickshank, Gitte S. Jensen

**Affiliations:** 1 NIS Labs, 807 St. George St., Port Dover, ON N0A 1N0, Canada; yuliuliu1995@outlook.com (L.Y.); dina@nislabs.com (D.C.); 2 NIS Labs, 1437 Esplanade, Klamath Falls, OR 97601, USA; ifeanyi@nislabs.com

**Keywords:** anti-inflammatory, CD25, CD69, interleukin-6, interleukin-13, immune modulation, interferon-gamma, monocytes, NK cells, surveillance, T cells

## Abstract

Immune protection associated with consuming colostrum-based peptides is effective against bacterial and viral insults. The goal for this study was to document acute changes to immune surveillance and cytokine levels after consuming a single dose of a nutraceutical blend in the absence of an immune challenge. A double-blind, randomized, placebo-controlled, cross-over pilot study involved healthy participants attending two clinic visits. Blood draws were performed pre-consumption and at 1, 2, and 24 h after consuming a blend of bovine colostrum- and hen’s egg-based low-molecular-weight peptides (CELMPs) versus a placebo. Immunophenotyping was performed by flow cytometry, and serum cytokines were measured by multiplex cytokine arrays. Consumption of CELMPs triggered increased immune surveillance after 1 h, involving monocytes (*p* < 0.1), natural killer (NK) cells (*p* < 0.1), and natural killer T (NKT) cells (*p* < 0.05). The number of NKT cells expressing the CD25 immunoregulatory marker increased at 1 and 2 h (*p* < 0.1). Increased serum levels of monocyte chemoattractant protein-1 (MCP-1) was observed at 2 and 24 h (24 h: *p* < 0.05). Selective reduction in pro-inflammatory cytokines was seen at 1, 2, and 24 h, where the 2-h reduction was highly significant for IL-6, IFN-γ, and IL-13. The rapid, transient increase in immune surveillance, in conjunction with the reduced levels of inflammatory markers, suggests that the CELMP blend of natural peptides provides immune benefits of use in preventive medicine. Further studies are warranted in chronic inflammatory conditions.

## 1. Introduction

Effective immune protection relies on constant surveillance activity where immune cells monitor the body for pathogens and transformed, virally infected, and malignant cells. In this process of concerted communication, the gut mucosal barrier plays a vital role in helping our immune system to receive and interpret signals and communicate with the rest of the body. Macrophages and dendritic cells in the mucosal surface sample antigens produced by the gut microbiome and ingested materials [1]. The downstream cellular communication within the mucosal tissue includes NK cells and T cells present in the lamina propria and Peyer’s Patches deeper in the tissue. Subsequently, the vagus nerve manages gut-to-brain communication, followed by communication between the central and peripheral nervous systems, to rapidly regulate immune surveillance processes [2].

Consumption of immune-modulating nutraceuticals supports natural immune surveillance processes and offers a unique opportunity for the use of botanical and natural food-based extracts. Using well-controlled study parameters, minute and often transient changes to immune biomarkers after consuming a single dose of immune-modulating natural products have been documented. Depending on the consumable nutraceutical, and in light of epigenetic reprogramming of immune cells through ‘immune training’ [3], it is possible that some biomarkers remain altered for a longer time. Previous research showed rapid and transient increases in immune surveillance by NK and T cells and changes to cytokine levels after consuming a low-molecular-weight peptide-rich extract from bovine colostrum, where effects were seen at 1 and 2 h after consumption of a single dose of 150 mg extract [4]. Based on the previous documentation of significant changes within 1–2 h, we selected a similar low-molecular-weight peptide blend to explore longer lasting effects.

The immune-modulating effects of low-molecular-weight peptides, naturally occurring in mammals and birds, involve molecular communication mechanisms to protect their offspring by transferring immune protection from the mother’s established and educated immune system to the offspring [5]. Newborn mammals receive a plethora of low-molecular-weight peptides by feeding on colostrum, which is a yellow liquid produced by the mother after birth. For avians, peptides conveying immune protection are already present in the unfertilized egg as a method of transferring immunity from the mother bird to the developing embryo. The immune-protective effects of such peptides are not species-specific, so peptides from mammalian or avian species can protect other species [6], and peptides produced by one individual can be absorbed orally by another individual [7,8].

The low-molecular-weight peptides in colostrum are diverse, and the proteomic profile differs from that of mature milk [9]. The peptides include proline-rich peptides, transfer factors, and growth factors [10]. Proline-rich peptides contain 25% proline amino acids and have unique immune-regulating properties concerning Th1/Th2 cytokine induction [11] and support of cytotoxic T cell function [12]. Transfer factors [13] comprise antigen-recognizing molecules with distinct roles in T-cell-mediated immune responses [14], specifically for the cytotoxic response, where some types of transfer factors tag transformed and virally infected cells for destruction, assisting cytotoxic T cells in recognizing and killing virally infected cells [15]. The peptides capable of transferring immune protection from one individual to another have been described as ‘transfer factors’; however, the nomenclature for transfer factors has changed over the years and was originally used to describe an extract from white blood cells [5,6]. Today, when discussing consumable nutraceutical products, the term transfer factors is often used to describe a more generic and less well-defined natural mixture of low-molecular-weight peptides, including lactoferrin, defensins, and cathelicidins [16]. Low-molecular-weight peptides in egg yolk also include cathelicidins [17], lysozymes, and ovotransferrins [18].

The low-molecular-weight peptides from bovine colostrum versus egg have differential effects on epithelial integrity and tight junctions when challenged with different types of bacteria [19], suggesting that consuming a blend of both sources of peptides may be beneficial to health in various ways. Other research teams have documented the immune benefits of consuming bovine colostrum low-molecular-weight peptides, including a murine study showing significant improvements in phagocytosis, TNF-α and IL-2 secretion, antibody formation, and NK cell cytotoxic activity after 7 days of consumption [20]. Several rodent studies showed similar immune benefits when consuming a blend of bovine and avian peptides [21,22]. The effect of bovine colostrum peptides on natural-killer-cell-mediated killing of target cells has been documented in vitro [23]. Safety of bovine colostrum ultrafiltrate has been recognized [24].

The clinical feasibility study reported here involved consumption of a nutraceutical blend of bovine colostrum- and hen’s egg-based low-molecular-weight peptides (CELMPs). This study examined changes to immune cell surveillance and activation status, as well as communication through cytokines and chemokines. The study was conducted on healthy adults, who were tested following an established placebo-controlled, randomized, double-blinded, cross-over study design [4,25], where each participant served as their own control. The cross-over design is the optimal method of ensuring paired data with respect to age, body mass index, diet, and lifestyle factors. As an extension of previously published studies following this design, follow-up visits and blood draws were performed the following morning to determine whether all biomarkers returned to the baseline after a full diurnal cycle. Based on a previous exploratory pilot study of a similar nature [26], this study focused on a small population but on a broad set of biomarkers. Documenting rapid changes to immune markers is not trivial, since the immune system undergoes circadian changes and is also affected by stress [27,28,29]. This clinical feasibility study was performed based on the need to explore whether rapid changes to immune markers were transient and returned to the baseline after 24 h or whether some biomarkers remained altered for longer time.

## 2. Materials and Methods

### 2.1. Study Design

**Figure 1:**
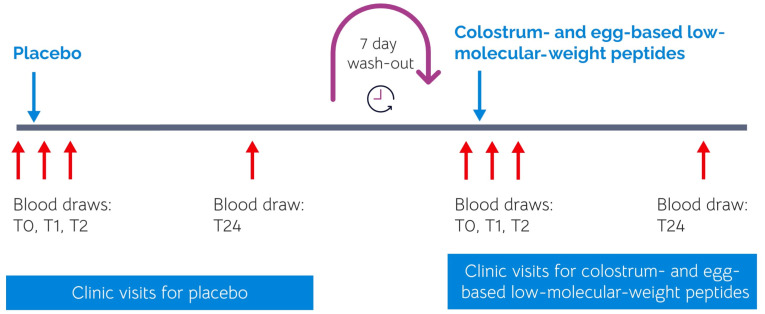
Diagram showing the involvement where all study participants were tested on two different clinic days, with blood draws at the baseline (T0), 1 h (T1), 2 h (T2), and 24 h (T24) after consuming a test product. An additional blood draw was performed 24 h after product consumption for both clinic days. The red arrows represent blood draw times as part of the core study design, at the baseline (T0), 1 h (T1), and 2 h (T2), and at 24 h after consuming a test product. For each sample collection time point, saliva was also collected (passive drool).

A randomized, double-blind, placebo-controlled, cross-over study design was used for this clinical proof-of-concept study (clinical trial registration NCT05364710), which was conducted in accordance with the Declaration of Helsinki and approved by the Ar-gus Independent Review Board, Tucson, AZ, USA (IRB approval number NIS 058-007). The study was carried out at NIS Labs, Oregon, USA. Based on a similar previous pilot study [26], four people were enrolled in the study after signing written informed consent (Table 1), as approved by the institutional review board of Argus IRB Inc., and completed the requirements of study participation (Figure 1). The participants consumed the active product and placebo 1–2 weeks apart in randomized order. 

The screening process included an interview to record age, body mass index (BMI), medical/surgical history, diet and lifestyle, current health status, medication, and use of nutritional supplements. The inclusion criteria were as follows: healthy adult people of either gender, age 18–75 years (inclusive), body mass index (BMI) between 18.0 and 34.9 kg/m^2^ (inclusive), veins easily accessible for the multiple blood draws, and willingness to comply with requirements: maintaining a consistent diet and lifestyle routine throughout the study, consistent habit of bland breakfasts on days of clinic visits, abstaining from exercising and nutritional supplements on the morning of a study visit, and remaining in an unexcited and calm state of mind during each visit. It was part of the screening procedure to ask nicotine users whether abstaining from nicotine for one hour before the clinic visit and during the clinic hours would cause them stress—if so, they could not participate. Participants were instructed to consume only water during the visits and encouraged to consume as much as they can to stay hydrated for the multiple blood draws.

The following exclusion criteria were used during screening: previous major gastrointestinal surgery; taking anti-inflammatory medications on a daily basis; currently in intensive athletic training; cancer during the past 12 months; chemotherapy during the past 12 months; current treatment with immune-suppressant medication; diagnosis with autoimmune disorder; donation of blood during the study or within the 4 weeks prior to study start; a cortisone injection within the past 12 weeks; vaccination during the last month; current use of antipsychotic, hypnotic, or anti-depressant prescription medication; ongoing acute infections; participation in another clinical trial study during this trial involving an investigational product or lifestyle change; an unusual sleep routine; unwillingness to maintain a constant intake of supplements over the duration of the study; anxiety about having blood drawn; pregnancy, nursing, or trying to become pregnant; and known food allergies related to ingredients in the active test product or placebo. People who met these inclusion and exclusion criteria were enrolled into the study after providing written informed consent. 

The clinic visits were scheduled at the same time of the day during the morning hours of 7:00–11:00 a.m., where participants arrived in a staggered manner (7:00 a.m., 7:15 a.m., 7:30 a.m., and 7:45 a.m.), and blood draws performed according to a strict schedule to minimize the effect of circadian fluctuations [27,28,29]. One of the two clinic visits involved consuming the active test product, and a second clinic visit involved consuming the placebo, and the results from the placebo-visit served as a control for the circadian variations in cytokine levels and immune surveillance for each participant. The interference of exercise [30] and stress [31,32,33] with the release versus homing of lymphocytes is well documented, and the clinical environment was managed to minimize physical and mental stress during each visit. Participants were instructed to remain calm and inactive for 3 h, comfortably seated in a chair in a private room. At one hour after a person’s arrival, the baseline blood sample was drawn. Immediately after the baseline blood draw, an encapsulated test product was provided with water and consumed in the presence of clinic staff. Blood samples were drawn 1 and 2 h after consumption of the test product. The same time schedule was followed for both visits for a given participant. 

Blood draws were performed using 23-gauge butterfly needles (BD Vacutainer Safety-Lok blood collection set). For each blood draw, 6 mL of blood was drawn into heparinized vacutainer tubes and 8 mL into serum separator tubes. The heparinized blood samples were used for immunostaining within the hour of each blood draw. The blood in the serum separator tubes was allowed to coagulate for 30–60 min at room temperature, after which the tubes were centrifuged for 15 min at room temperature. The serum was harvested and banked at −80 °C for subsequent testing of the cytokine profile. 

### 2.2. Reagents

Heparin vacutainer tubes, serum separator tubes, butterfly needles, and the mono-clonal antibody CD25-Brilliant Violet 421 (clone 2A3) were purchased from Becton-Dickinson (Franklin Lakes, NJ, USA). The monoclonal antibodies CD3-SuperBright702 (clone OKT3), CD56-Phycoerythrin (clone CMSSB), and CD69-Fluorescein isothiocyanate (clone FN50), flow cytometer performance tracking beads, wash and shut-down solutions, de-bubble buffer, and High Yield Lysing buffer™ were purchased from Thermo Fisher Scientific (Waltham, MA, USA). Customized Bio-Plex Pro™ human cytokine arrays were purchased from Bio-Rad Laboratories Inc. (Hercules, CA, USA). 

### 2.3. Consumable Test Products

The active test product, Transfer Factor Tri-Factor (TF Tri-Factor), was obtained from the distributor 4Life Research, LLC, Sandy, UT, USA. One dose of the product contains 600 mg of a blend of three extracts: (1) ultra-filtered peptides from bovine colostrum, (2) a concentrate of nano-filtered bovine colostrum, and (3) a concentrate of peptides from chicken egg yolk. The placebo consisted of rice flour encapsulated in similar capsules as the active test product.

### 2.4. Immune Cell Evaluation by Flow Cytometry

Triplicate samples of 100 µL heparinized whole blood were stained with fluorochrome-conjugated monoclonal antibodies directed at the cell surface markers CD3, CD25, CD56, and CD69 and incubated for at least 15 min at room temperature in the dark, followed by addition of 2 mL of High-Yield Lysing buffer, mixing by pipetting and vortexing, and incubation for at least 10 min at room temperature in the dark to allow lysing of the erythrocytes. Samples were transferred to 2 mL deep-well 96-well plates and analyzed by flow cytometry within 4 h of staining. Samples were acquired using an acoustic-focusing Attune™ Nxt flow cytometer (Thermo Fisher Scientific, Waltham, MA, USA) with a microplate-based Autosampler. Data analysis utilized gating on forward/side scatter to identify lymphocyte and monocyte populations, followed by gating based on the CD56 NK cell marker and the CD3 T cell marker, to identify the CD3-CD56+ NK cells, CD3+CD56+ NKT cells, and CD3+ CD56- T cells. Each cell subset was then analyzed for expression levels of the immunoregulatory markers CD25 and CD69.

### 2.5. Serum Levels of Cytokines, Chemokines, and Growth Factors

Serum samples from all blood draws were used for the evaluation of changes to blood levels of 27 cytokines and chemokines using Bio-Plex magnetic bead protein arrays (Bio-Rad Laboratories Inc.) and xMAP technology (Luminex, Austin, TX, USA). The following markers were tested: IL-1β, IL-1ra, IL-2, IL-4, IL-5, IL-6, IL-7, IL-8, IL-9, IL-10, IL-12 (p70), IL-13, IL-15, IL-17, Eotaxin, Basic FGF, G-CSF, GM-CSF, IFN-γ, IP-10, MCP-1 (MCAF), MIP-1α, MIP-1β, PDGF-BB, RANTES, TNF-α, and VEGF. The 27 types of magnetic beads (one type for each analyte) contain specific fluorescence signatures for each bead type, are pre-coated with capture antibodies directed at the 27 cytokines, and were mixed, allowing for simultaneous quantification of the 27 cytokines. The testing of serum cytokine levels was performed in 96-well plates by adding magnetic beads to serum samples and incubating for 60 min, after which the beads were washed on a magnet to remove unbound serum proteins. The samples were treated with biotinylated detection antibodies towards the 27 cytokines, incubated for 45 min, and washed to remove unbound detection antibodies. Streptavidin-PE was added to the samples and incubated for 10 min, whereafter the beads were washed to remove unbound streptavidin-PE. The fluorescence intensity of the beads was recorded using a MagPix microplate reader, and the mean fluorescence intensity of the analytes in each sample was calculated using the xPonent software (Version 4.2, Luminex, Austin, TX, USA).

### 2.6. Saliva Secretory IgA

After each blood draw, each participant was instructed to collect a small sample of unstimulated saliva using a SalivaBio saliva collection aid for passive drool. The samples were frozen at −20 °C immediately after collection. The samples were shipped frozen to Salimetrics, LLC (Carlsbad, CA, USA) for testing of salivary secretory IgA (sIgA).

### 2.7. Statistical Analysis

The data analysis involved calculation of changes from the baseline to later blood draws on the day each participant consumed the active product, in context of the person’s circadian changes on the day they consumed the placebo. Changes to biomarkers were computed as average and standard deviation for each data set using Microsoft Excel (Version 2404, Microsoft Corporation, Redmond, WA, USA). Post-consumption changes from the baseline to 1 h and 2 h were evaluated by between-treatment analysis for each time point. The evaluation used within-subject analysis and the two-tailed paired *t*-test, where statistical significance was set to *p* < 0.05 and a high level of significance to *p* < 0.01.

## 3. Results

### 3.1. Immune Surveillance

**Figure 2:**
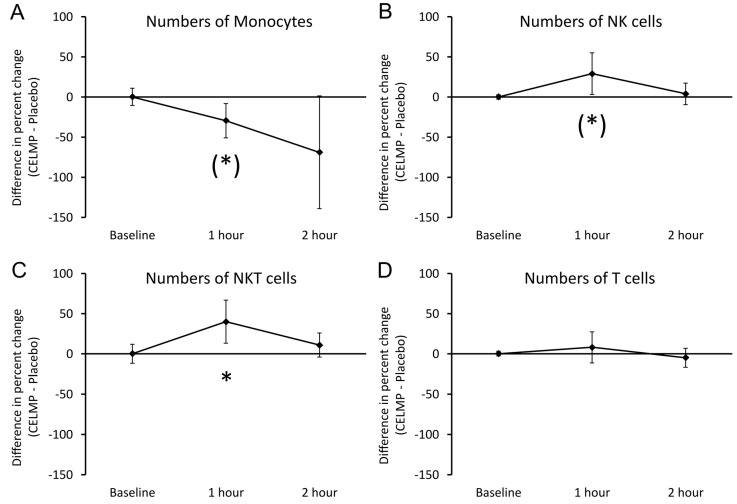
Differences in changes to immune cell trafficking reflected by the numbers of immune cells in the blood circulation within 2 h after consumption of CELMPs versus the placebo. The results are shown as the group averages ± standard error of the mean of the individual percent changes from the baseline after consuming CELMPs, where changes after consuming the placebo are subtracted. (**A**) Monocyte numbers: A gradual reduction was seen at 1 and 2 h, and the reduction at 1 h reached a statistical trend. (**B**) NK cell numbers: An increase was seen at 1 h, returning to similar levels as after consuming the placebo at 2 h. The increase at 1 h reached a statistical trend. (**C**) NKT cell numbers: An increase after consuming the CELMPs compared to the placebo reached statistical significance at 1 h, returning to similar levels to after consuming the placebo at 2 h. (**D**) Numbers of T cells: No significant differences were observed in the T cell numbers after consuming CELMPs compared to the placebo. Levels of statistical significance are shown in the graphs, where changes from the baseline to a later time point are indicated by asterisks: *p* < 0.10: (*), *p* < 0.05: *.

Consumption of CELMPs triggered rapid changes to immune surveillance, as evidenced by rapid changes to the numbers of specific types of immune cells in the blood circulation (Figure 2). At 1 h after consumption of a single dose of 600 mg, the difference in the number of circulating monocytes (Figure 2A) was lower than at 1 h after consuming the placebo, reaching a statistical trend (*p* < 0.1), suggesting an increased trafficking of monocytes into tissue. After 2 h, the average level of monocytes in the blood circulation continued to decrease but was no longer statistically significant compared to after consuming the placebo. Both NK cells and NKT cells increased within the same 1-h interval after consuming CELMPs compared to changes after consuming the placebo. The increased levels of CD3-CD56+ NK cells (Figure 2B) reached a statistical trend at 1 h (*p* < 0.1). The increased levels of CD3+CD56+ NKT cells (Figure 2C) reached statistical significance at 1 h (*p* < 0.05), suggesting that NKT cells were mobilized from tissue. In contrast, the numbers of T cells (Figure 2D) did not show different changes after consuming the placebo.

### 3.2. Increased Numbers of Immune Cells Expressing CD25 but Not CD69

**Figure 3:**
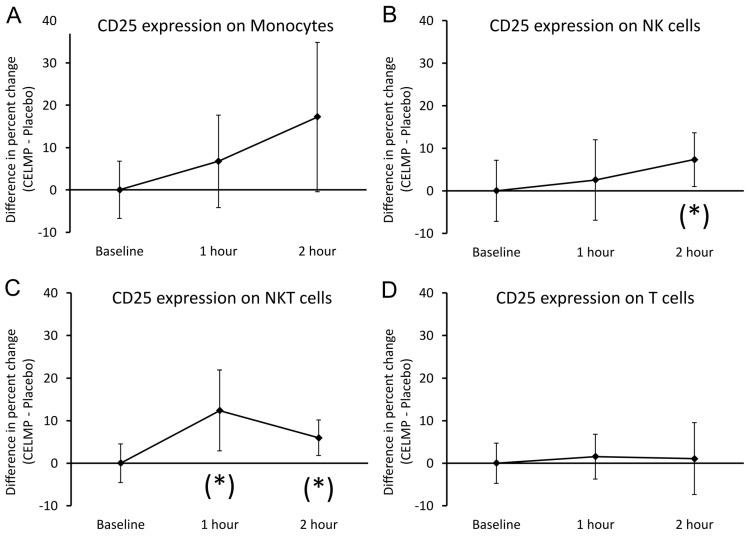
Differences in changes to expression of the immunoregulatory marker CD25 on 4 types of immune cells in the blood circulation within 2 h after consumption of CELMPs versus the placebo. The results are shown as the group averages ± standard error of the mean of the individual percent changes from the baseline after consuming CELMPs, where changes after consuming the placebo are subtracted. (**A**) CD25 expression on monocytes: A gradual increase was seen at 1 and 2 h, not reaching statistical significance. (**B**) CD25 expression on NK cells: A gradual increase was seen at 1 and 2 h, reaching a statistical trend at 2 h. (**C**) CD25 expression on NKT cells: An increase after consuming the CELMPs compared to the placebo reached statistical trends at 1 and 2 h. (**D**) CD25 expression on T cells: No significant differences were observed after consuming CELMPs compared to the placebo. Levels of statistical significance are shown in the graphs, where changes from the baseline to a later time point are indicated by asterisk: *p* < 0.10: (*).

The expression of the immunoregulatory marker CD25 was evaluated on monocytes, NK cells, NKT cells, and T cells (Figure 3). The expression of CD25 on monocytes (Figure 3A) showed a gradual increase over the 2 h after consumption, but the difference in CD25 expression after consuming CELMPs compared to the placebo did not reach statistical significance. The expression of CD25 on CD3-CD56+ NK cells (Figure 3B) showed a gradual increase over the 2 h after consumption, reaching a statistical trend at 2 h. The expression of CD25 on CD3+CD56+ NKT cells (Figure 3C) was higher at 1 h after consuming CELMPs than after consuming the placebo, and the difference reached a statistical trend (*p* < 0.1). Though a gradual decrease was seen at 2 h, the statistical trend (*p* < 0.1) remained. In contrast, the expression of CD25 on T cells (Figure 3D) was similar after consuming CELMPs and after consuming the placebo.

**Figure 4:**
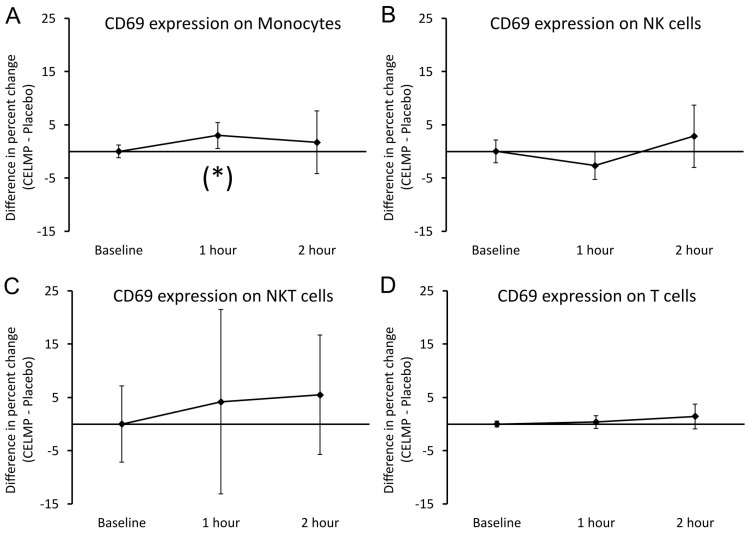
Differences in changes to expression of CD69 on 4 types of immune cells in the blood circulation within 2 h after consumption of CELMPs versus the placebo. The results are shown as the group averages ± standard error of the mean of the individual percent changes from the baseline after consuming CELMPs, where changes after consuming the placebo are subtracted. (**A**) CD69 expression on monocytes: A gradual increase was seen at 1 h, reaching statistical trend and returned to similar levels to after consuming the placebo at 2 h. (**B**) CD69 expression on NK cells: No significant differences were observed after consuming CELMPs compared to the placebo. (**C**) CD69 expression on NKT cells: No significant differences were observed after consuming CELMPs compared to the placebo. (**D**) CD69 expression on T cells: No significant differences were observed after consuming CELMPs compared to the placebo. Levels of statistical significance are shown in the graphs, where changes from the baseline to a later time point are indicated by asterisk: *p* < 0.10: (*).

The expression of CD69 was evaluated on monocytes, NK cells, NKT cells, and T cells (Figure 4). The expression of CD69 on monocytes showed a very mild increase at 1 h, reaching a statistical trend (*p* < 0.1), and a return at 2 h to similar expression levels as after consuming the placebo. No significant differences in CD69 expression were seen between CELMPs and the placebo for the other three cell types: CD69 on CD3-CD56+ NK cells, CD3+CD56+ NKT cells, and T cells.

### 3.3. Decreased Pro-Activating Cytokine Production

**Figure 5:**
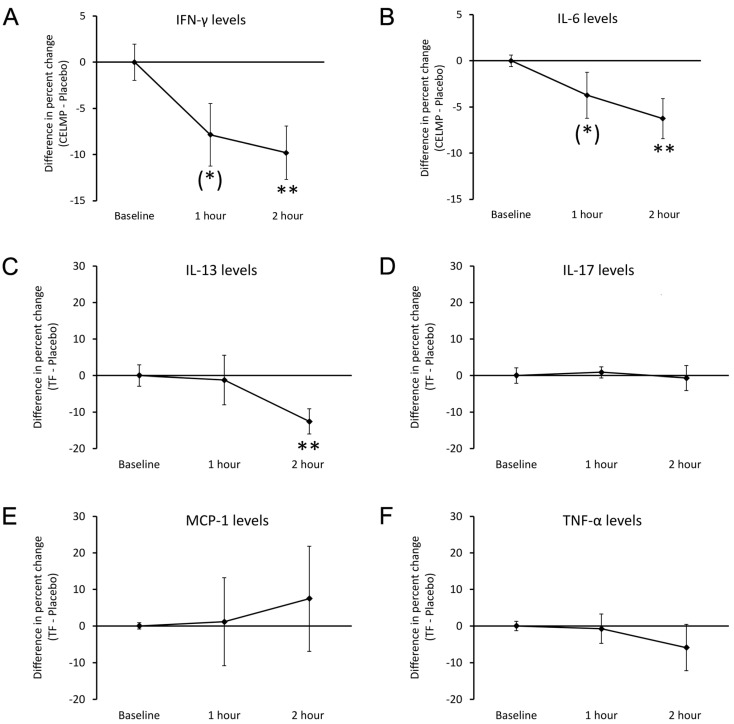
Differences in changes to cytokine levels in the blood circulation within 2 h after consumption of CELMPs versus the placebo. The results are shown as the group averages ± standard error of the mean of the individual percent changes from the baseline after consuming CELMPs, where changes after consuming the placebo are subtracted. (**A**) Interferon-gamma (IFN-γ) levels: A gradual decrease was seen at 1 and 2 h, reaching a statistical trend at 1 h and a high level of significance at 2 h. (**B**) Interleukin-6 (IL-6) levels: A gradual decrease was seen at 1 and 2 h, reaching a statistical trend at 1 h and a high level of significance at 2 h. (**C**) Interleukin-13 (IL-13) levels: A gradual decrease was seen at 2 h, reaching a high level of significance at 2 h. (**D**) Interleukin-17 (IL-17) levels: No significant differences were observed after consuming CELMPs compared to the placebo. (**E**) Monocyte chemoattractant protein-1 (MCP-1): An increase was observed at 2 h but did not reach statistical significance. (**F**) Tumor necrosis factor-alpha (TNF-α) levels: A gradual decrease was seen at 2 h but did not reach statistical significance. Levels of statistical significance are shown in the graphs, where changes from the baseline to a later time point are indicated by asterisks: *p* < 0.10: (*) and *p* < 0.01: **.

A highly selective change in cytokine levels was seen when comparing the changes after consuming CELMPs to changes after consuming the placebo (Figure 5). Four pro-inflammatory cytokines showed reduced levels after consuming CELMPs when compared to changes after consuming the placebo: The reduced levels of interferon-gamma (IFN-γ, Figure 5A) and interleukin-6 (IL-6, Figure 5B) reached a statistical trend at 1 h (*p* < 0.1) and a high level of statistical significance at 2 h (*p* < 0.01). The reduced levels of interleukin-13 (IL-13, Figure 5C) reached a high level of significance after 2 h, whereas the reduced level of tumor necrosis factor-alpha (TNF-α) did not reach statistical significance. To illustrate the selectiveness of the response, other pro-inflammatory cytokines, including interleukin-17 (IL-17, Figure 5D), did not show any differences between CELMPs and the placebo. There was a mild increase in monocyte chemoattractant protein-1 (MCP-1), possibly associated with the increased trafficking of monocytes, but the increase in MCP-1 did not reach statistical significance.

### 3.4. Immune Changes after 24 h

**Figure 6:**
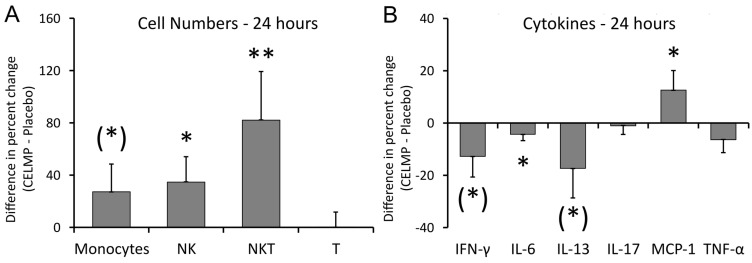
Differences in changes to immune markers in the blood circulation 24 h after consumption of CELMPs versus the placebo. The results are shown as the group averages ± standard error of the mean of the individual percent changes from the baseline after consuming CELMPs, where changes after consuming the placebo are subtracted. (**A**) The numbers of monocytes, NK cells, and NKT cells increased, whereas there were no changes to T cell numbers. The increased numbers of monocytes reached a statistical trend, the numbers of NK cells were significant, and the increased numbers of NKT cells were highly significant. (**B**) Cytokine levels: Four cytokines showed reduced levels: interferon-gamma (IFN-γ), interleukin-6 (IL-6), interleukin-13 (IL-13), and tumor necrosis factor-alpha (TNF-α). The reduced levels of IFN-γ and IL-6 reached a statistical trend, and the reduced level of IL-6 was mild but statistically significant. No change was seen for IL-17. In contrast, a statistically significant increase in monocyte chemoattractant protein-1 (MCP-1) was observed at 24 h after consumption. Levels of statistical significance in changes from the baseline to a later time point are indicated by asterisks: *p* < 0.10: (*), *p* < 0.05: *, and *p* < 0.01: **.

In addition to documenting the immediate changes at 1 and 2 h, an additional follow-up blood draw at 24 h served to document longer-lasting effects (Figure 6). Increases in the numbers of immune cells in the blood circulation were seen (Figure 6A). An increase in monocyte numbers reached a statistical trend, increased NK cell numbers were statistically significant (*p* < 0.05), and the increased numbers of NKT cells reached a high level of statistical significance (*p* < 0.01).

The longer-lasting effects on cytokine levels (Figure 6B) showed that the selective and relatively mild reduction in the four pro-inflammatory cytokines remained. The reduced levels of IFN-γ and IL-13 reached a statistical trend (*p* < 0.1), the mild reduction in IL-6 was statistically significant (*p* < 0.05), and the reduced levels of TNF-α did not reach statistical significance. The IL-17 level did not show any differences between CELMPs and the placebo. In contrast, the increased level of MCP-1 reached statistical significance at 24 h (*p* < 0.05).

### 3.5. Saliva Secretory IgA and IgG

Consumption of CELMPs did not show significant changes to saliva secretory IgA when compared to the placebo (Table 2).

## 4. Discussion

Immune surveillance is a key component of immune protection, as many types of immune cells traffic through blood into tissues and return via the lymphatic system. The circadian rhythm of this process is well described, and consuming many types of natural products has been shown to provide mild but highly recognizable support of immune surveillance [4,25]. The work reported here showed rapid effects on a blend of bovine and avian low-molecular-weight peptides (CELMPs) on the human immune system, along with a prolonged effect at 24 h. The effects included increased immune surveillance, with monocytes migrating into tissue at one hour. At one hour after consumption, there was already an increase in CD25-positive NK and NKT cells in the blood circulation, indicating mobilization of cells with increased alertness. There were also increased levels of CD25-positive monocytes in the blood circulation (not significant), which has been linked to increased maturity and phagocytic activity [34,35]. This was not seen for CD69, indicating a selective effect and suggesting that CELMPs triggered gut mucosal immune and vagus nerve activity and nerve signaling to peripheral immune tissue, leading to mobilization of CD25-positive NK and NKT cells into the blood circulation. The mild, highly selective increases reported here suggest that tissue-resident monocytes, NK cells, and NKT cells expressing CD25 entered the blood circulation to traffic to new areas as part of immune surveillance activity. The expression of the interleukin-2 receptor CD25 on NK cells makes the cells more responsive to interleukin-2, suggesting an increased state of responsiveness of these NK cells. CD25 is also increased on memory-like NK cells, which have a longer lifespan and increased interferon-gamma production [36]. It is important to differentiate this mobilizing effect in in vivo and in vitro studies on changes to CD25 expression. In vitro studies that evaluate effects of natural products on CD25 expression on immune cells typically use a 24-h incubation time, and in the limited environment of a cell culture there is no influx of CD25-positive cells; rather, the increase in CD25 expression is a result of immune cell activation in the cell culture [37,38].

Oral application of bovine colostrum low-molecular-weight peptides has been shown to provide rapid protection from bacterial and viral infectious challenges in a rodent model [38]. This suggested rapid alerting and recruitment of innate immune cells to mucosal barriers, prior to the immune challenge, but the underlying mechanisms were unknown. The consumption of CELMPs reported here was associated with rapid changes to blood levels of selected cytokines and chemokines. Specifically, the reduced levels of the pro-inflammatory cytokines IL-6, IL-13, and TNF-α suggest an anti-inflammatory effect, which was an unexpected effect of CELMP consumption and warrants further evaluation in preclinical and clinical studies. The effects were mild and within the spectrum of normal daily changes in the immune system that occur during the circadian cycle. It is possible that reduced inflammatory interference was associated with the homing of monocytes seen after 1 h, as the effect may have allowed monocytes to better sense chemoattractant signals from tissue. 

A rapid increase in the chemotactic marker monocyte chemoattractant protein-1 (MCP-1), while insignificant at 1 or 2 h, reached significance at 24 h (*p* < 0.05). Further work is needed to establish whether the increase in MCP-1 helped initiate the observed monocyte migration, resulting initially in a transient mild reduction in blood monocyte levels as they migrated into tissue, and followed by an increase in blood monocyte levels at 24 h (*p* < 0.1). Previous work from our team showed an increase in phagocytic activity of mononuclear and polymorphonuclear phagocytes after consumption of a single dose of bovine colostrum low-molecular-weight fraction [4]. 

The study also aimed to document effects involving the adaptive immune response, particularly in terms of secretory IgA (sIgA)-mediated mucosal immune protection. A previous study on consumption of a multivitamin containing CELMPs showed that there was an increase in saliva sIgA over time, reaching a high level of statistical significance at 4 weeks [39]. In this study, sIgA levels were evaluated, but no statistically significant changes were detected over the 2- or 24-h periods. This suggests that an increase in sIgA-mediated immune protection along mucosal membranes may be a cumulative effect from daily consumption of CELMPs over an extended period of time. It is unknown whether CELMPs affect the number or secretory activity of plasma cells adjacent to mucosal surfaces or whether they affect a more fundamental aspect of B cell proliferation and honing to mucosal tissue.

## 5. Conclusions

The increased immune surveillance reported here in the absence of an infectious insult to the immune system has multiple implications, including enhanced immune patrolling of tissue for potential pathogens and transformed cells. This, in conjunction with the reduced levels of inflammatory markers, shows that this increased immune alertness takes place in the absence of pro-inflammatory immune activation. Some effects were transient and had returned to the baseline after a full circadian cycle of 24 h; however, the increase in the numbers of natural killer cells and higher levels of MCP-1 in the blood circulation showed a longer-lasting effect. The present pilot study had limitations, primarily due to the small population size and the short follow-up duration of 24 h. Further work is warranted to elucidate the clinical implications of monocyte homing and regulation of inflammation and should include a larger group of participants and a longer follow-up duration. In addition, the immunophenotyping should be broadened to include evaluation of M1 versus M2 monocyte/macrophage polarization, as well as more in-depth characterization of natural killer types and the functional state of cytotoxic response to target cells. Clinical evaluation should also include long-term effects of CELMP consumption in populations with chronic mucosal and non-mucosal inflammatory disorders. Further research is in progress to document whether additional biomarkers are of value for documenting effects of nutraceutical products on the support of immune defense activity and epigenetic reprogramming during immune training, as well as a reduction in inflammation. 

## Figures and Tables

**Table 1:** Demographics of the study population.

Participant	Gender	Age	BMI
P001	Male	37.4	27.94
P002	Male	74.5	19.63
P003	Male	52.8	23.82
P004	Female	38.5	28.5
Average ± StDev		50.8 ± 17.3	25 ± 4.1
Range		37.4–74.5	19.63–28.5

**Table 2:** Saliva secretory IgA levels.

	N	Baseline ^a^	1 h ^a^	2 h ^a^	24 h ^a^
CELMPs	4	221.32 ± 38.18	129.64 ± 24.61	212.86 ± 42.34	225.67 ± 36.4
Placebo	4	287.27 ± 68.89	187.15 ± 28.28	203.32 ± 46.86	250.5 ± 56.85

^a^ The average ± standard error is shown in µg/mL.

## Data Availability

The data presented in this study are available on request from the corresponding author upon reasonable request.
